# Targeting HSF1 for cancer treatment: mechanisms and inhibitor development

**DOI:** 10.7150/thno.82431

**Published:** 2023-04-17

**Authors:** Yeh Chin, Khanisyah E Gumilar, Xing-Guo Li, Brahmana A. Tjokroprawiro, Chien-Hsing Lu, Jianrong Lu, Ming Zhou, Robert W. Sobol, Ming Tan

**Affiliations:** 1 Graduate Institute of Biomedical Sciences and Research Center for Cancer Biology, China Medical University, Taichung, Taiwan, R.O.C.; 2 The Department of Obstetrics and Gynecology, Medical Faculty, Universitas Airlangga, Surabaya, Indonesia.; 3 Department of Gynecology and Obstetrics, Taichung Veterans General Hospital, Taichung, Taiwan, R.O.C.; 4 Department of Biochemistry and Molecular Biology, College of Medicine, University of Florida, Gainesville, USA.; 5 Cancer Research Institute and School of Basic Medical Sciences, Central South University, Changsha, China.; 6 Department of Pathology and Laboratory Medicine, Warren Alpert Medical School & Legorreta Cancer Center, Brown University, Providence, USA.; 7 Institute of Biochemistry & Molecular Biology, China Medical University, Taichung, Taiwan, R.O.C.

**Keywords:** HSF1, Heat shock response, cellular stress, cancer

## Abstract

Heat Shock Factor 1 (HSF1) is a master regulator of heat shock responsive signaling. In addition to playing critical roles in cellular heat shock response, emerging evidence suggests that HSF1 also regulates a non-heat shock responsive transcriptional network to handle metabolic, chemical, and genetic stress. The function of HSF1 in cellular transformation and cancer development has been extensively studied in recent years. Due to important roles for HSF1 for coping with various stressful cellular states, research on HSF1 has been very active. New functions and molecular mechanisms underlying these functions have been continuously discovered, providing new targets for novel cancer treatment strategies. In this article, we review the essential roles and mechanisms of HSF1 action in cancer cells, focusing more on recently discovered functions and their underlying mechanisms to reflect the new advances in cancer biology. In addition, we emphasize new advances with regard to HSF1 inhibitors for cancer drug development.

## Introduction

Heat Shock Factor 1 (HSF1) is a master regulator of cellular stress responsive signaling. Traditional roles of HSF1 relate to its function, as a transcription factor, to control transcription of a large family of heat shock proteins (HSPs). These heat shock proteins are responsible for protecting cells from proteotoxic stress-induced damage, such as heat shock 1, 2. Emerging evidence also suggests that HSF1 regulates a non-heat shock responsive transcriptional network to respond to metabolic, chemical, and genetic stress. In the last decade, important roles for HSF1 in cellular transformation and cancer development have been extensively studied. HSF1 supports malignant transformation by modulating multiple pathways regulating cellular proliferation, cell survival, protein synthesis and cellular metabolism 3-6. Our laboratory, and others have shown that HSF1 can modulate glucose metabolism, and mediates tumorigenesis, cell proliferation and drug resistance in cancer cells 3, 7-10. Importantly, while HSF1 has been demonstrated to be critical in oncogenesis, knockout of HSF1 has only modest effects in mice and flies under non-stressed conditions, supporting a potential therapeutic window. Due to its importance in coping with stressful conditions and survival, research on HSF1 has been very active. New functions and molecular mechanisms underlying these functions have been continuously discovered, suggesting new targets for novel cancer treatment strategies.

In this article, we will review the essential roles and mechanisms of HSF1 in response to heat shock and non-heat shock stress conditions, emphasizing recently discovered functions and their underlying mechanisms to reflect the new advances in the biology of HSF1. In addition, we discuss advancements towards the development of HSF1 inhibitors for cancer treatment.

## HSF1 family proteins and their structures

Functional proteins need to maintain correct structural folds and accurate complex assembly. In response to a proteostasis imbalance, folded protein intermediates, especially those with complicated domains, may expose hydrophobic amino acid residues and induce protein misfolding and aggregation 11. To avoid such negative outcomes, the heat shock response (HSR) is activated and serves as a protective mechanism to refold or degrade the misfolded intracellular proteins that accumulate under stressful cellular conditions. The activation of this stress response network helps to maintain the stability of the properly folded proteins as a strategy to cope with cellular stress 2, 12.

HSF1 is the master regulator of the heat shock response. It functions by binding to the heat shock element (HSE), an upstream promoter region in HSR genes, and facilitates transcriptional activation of HSPs to initiate the HSR. HSF1 is expressed constitutively in most tissues and cell types, but is inert or inactive under non-stress conditions 13. Once the stress-inducible HSF1 is triggered or activated by such extreme proteotoxic conditions (e.g., heat shock, oxidative stress, toxins, heavy metals, amino acid analogues, bacterial infections, and various small-molecule pharmacologically active substances), HSPs, acting as chaperones, are synthesized to prevent protein misfolding and aggregation 13, 14. Therefore, HSF1 plays a central role in regulating cellular proteostasis.

On the contrary, in cancer cells, HSF1 is constitutively activated and its target genes are avidly expressed. HSF1 could assist cancer cells to survive and evade programmed cell death, which leads to enhanced oncogenesis and tumor progression 5, 6, 15-17. Given that HSF1 overexpression is related to the poor outcome in various solid tumors, HSF1 could serve as a potential prognostic biomarker to predict progression toward more malignant phenotypes, such as metastasis 18, 19. On the other hand, because HSF1 is a key factor of tumorigenesis, developing specific drugs to inhibit HSF1 activity is a potential therapeutic agent 13, 18, 20.

**Figure 1:**
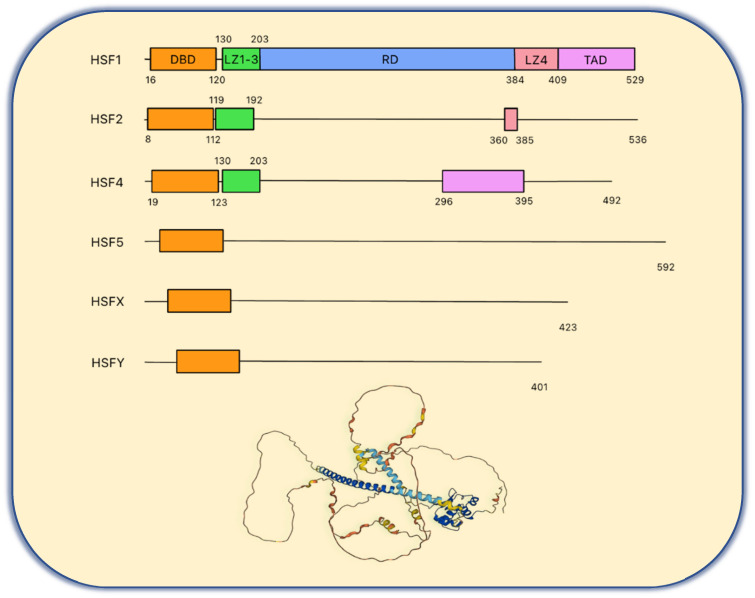
** The basic structure of human HSF family proteins.** The basic structure of human HSF family proteins and the HSF1 protein 3D structure from the Alphafold protein structure database (https://alphafold.ebi.ac.uk/entry/Q00613). DBD, DNA binding domain; LZ1-3, leucine zipper 1-3; RD, regulatory domain; LZ4, leucine zipper 4; TAD, transactivation domain.

There are six heat shock transcription factors encoded in the human genome **(Figure 1)**, including HSF1, HSF2, HSF4, HSF5, HSFX and HSFY. HSF3 has been identified in mice but not in humans 21. HSF2 is highly expressed in the testis and during the early stage of development and plays a limited role in HSR 22, 23. However, recent studies have shown that HSF2 interacts with HSF1 and shares similar chromatin occupancy sites with HSF1 to drive tumorigenesis in the support of malignancy 24. Moreover, treating cells with bortezomib, a proteasome inhibitor, will induce HSF2 transcription that is enhanced by HSF1 25. Together, these studies reveal a new interaction and pharmacological modulation between HSF1 and HSF2.

HSF4 is crucial during the eye lens development and its mutations are related to cataract 26. It was also found that HSF4 controls lens differentiation via *p53* cell death pathways 27. HSF5 is not a well-known factor among the HSF family members. However, in lung adenocarcinoma, it has been shown that HSF5 plays a regulatory role in immune infiltration, suggesting that this isoform may serve as a potential immunotherapy target 28. HSFX and HSFY are located on the X chromosome and Y chromosome, respectively. Although the function of HSFX is not clear, HSFY is expressed in the testis and loss of HSFY causes male infertility 29.

As shown in the **Figure 1**, the chief functional domain of HSF1 resides in the N-terminal DNA binding domain (DBD), which binds to the HSE comprised of three inverted GAA repeats once the HSR is triggered and the HSF1 homotrimer forms 30. Biochemical analysis has revealed that HSF1 trimerization is due to the leucine zipper oligomerization domain (LZ1-3), followed by the DBD and a flexible linker. LZ1-3 is characterized by its amphiphilic helix with the hydrophobic heptad repeated (HR-A/B) and forms the trimer as a coiled-coil structure via the intermolecular interaction during HSR 22, 30, 31. Under non-stress conditions, HSF1 remains as an inactive monomer due to an intramolecular interaction between LZ1-3 and LZ4 (HR-C) to avoid formation of the active trimer 31. The regulatory domain (RD) is located between LZ1-3 and LZ-4. Of note, there are many amino acid residues that are the target of post translational modifications (PTMs) in HSF1 that give rise to diverse regulatory mechanisms 31, 32. The last functional region is the C-terminal transactivation domain (TAD). It was found that deletion of TAD leads to cell death during heat shock. Thus, HSF1 with TAD is vital to the survival of cells under stressful conditions 33. HSF2 and HSF4 also have DBD domains and LZ domains, but the structures of these two proteins are complicated 22, 30, 31.

## The regulatory mechanism of HSF1

### Activation and inactivation of HSF1

**Figure 2:**
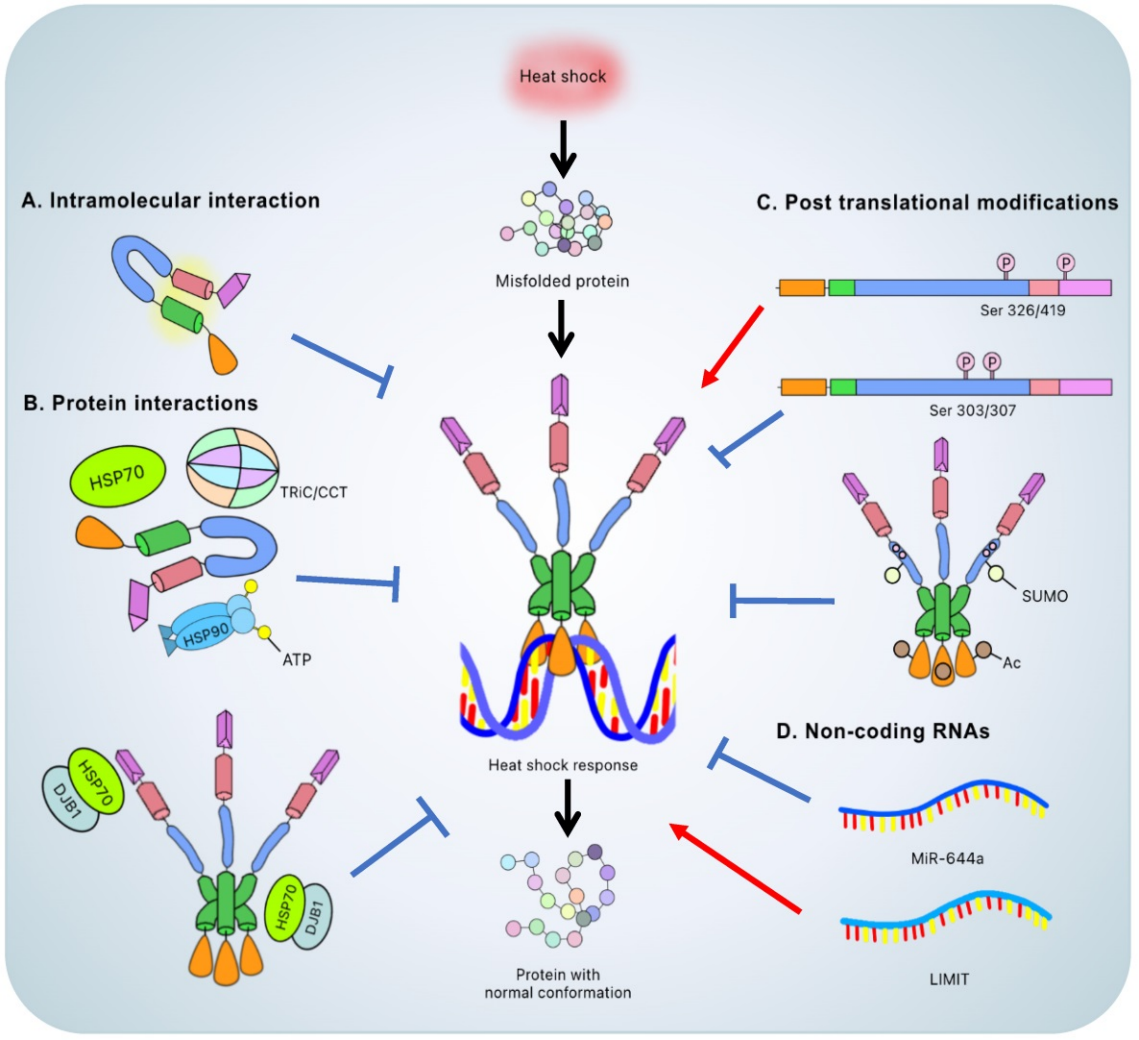
** The heat shock response and the regulation of HSF1**. The heat shock response is induced by the HSF1 activated trimer binding to the heat shock element that leads to resolution of the heat shock induced stress and accurate protein conformation. The methods to regulate the HSF1 include: **A.** intramolecular interaction between LZ1-3 and LZ4 to keep the inert monomeric state; **B.** interaction with other proteins, such as ATP-binding ''closed'' HSP90, HSP70 and TRiC/CCT to keep the inert monomeric state or interaction between HSP70-DnaJB1 complex and LZ1-3 or TAD to attenuate the heat shock response; **C.** post translational modification such as phosphorylation of Ser326/419 and Ser303/307 considered to be the activated and inactivated signals, respectively; acetylation of DBD and phosphorylation-dependent SUMOylation of RD to attenuate the heat shock response and **D.** non-coding RNA to inactivate and activate the heat shock response via miR-644a and LIMIT, respectively. **Color representation:** DNA-binding domain (orange); leucine zipper 1-3 (green); regulatory domain (blue); leucine zipper 4 (pink); transactivation domain (purple).

Without cellular stresses, HSF1 is suppressed by the LZ1-3/LZ4 interaction and kept in the cytoplasm by cytosolic chaperones (HSP90, HSP70, HSP40) and chaperonins (TRiC/CCT) 22, 34. HSF1 dissociates from the HSPs in response to a variety of stresses, including heat shock, heavy metal exposure, oxidants, and proteotoxic agents, allowing it to convert the monomer into a DNA binding homotrimer and allow translocation into the nucleus 13, 14. The HSF1 trimer binds to the HSE and regulates the expression of HSR genes, reducing cell stress and protecting the cell from stress-induced cell death. Thus, trimerization is an important step in activating HSF1 and allowing the DBDs to bind to the HSE 13, 31 (**Figure 2**).

According to an *in vitro* model, the ascending temperature is the primary inducer of HSF1. LZ1-3 and LZ4 act as intrinsic regulators, assisting HSF1 in displaying the monomeric inactive state via an intramolecular interaction. Both domains exhibit temperature-dependent conformational changes. As the temperature rises, the unfolding-refolding equilibrium between LZ1-3 and LZ4 shifts to unfolding, resulting in trimer formation and HSF1 activation 35. However, HSF1 activation, at lower environmental temperatures, was also reported while expressing human HSF1 in Drosophila cells 36. Our results also showed that oncogene ErbB2 overexpression leads to HSF1 activation 7. These results indicate that the mechanism of HSF1 activation could be temperature independent, complicating the model.

HSF1 interacts with molecular chaperones, undergoes a variety of PTMs 31, 37, and is influenced by regulatory non-coding RNAs 38 to mediate regulatory mechanisms. Furthermore, under pathological conditions, endogenous factors dysregulate HSF1 and cause a malfunction of various cellular processes. For example, loss of FBXW7, a Skp1-Cul1-F box ubiquitin ligase complex protein, induces HSF1 accumulation and enhances metastasis in melanoma since HSF1 drives a metastasis-promoting transcriptional program 39. As a result, studying the regulatory mechanisms in HSF1 under normal and abnormal conditions could be a promising area for the development of novel therapeutic agents.

HSF1 will be blocked if HSPs build up since these chaperones will form complexes with HSF1. This counter feedback mechanism prevents excessive HSR activation 40. Furthermore, PTMs on specific HSF1 residues, such as Ser303/Ser307 phosphorylation or K80 acetylation, turns off the HSR 31. In summary, under stressful conditions, the inhibitory regulation of HSF1 by chaperones and PTMs maintains an appropriate balance.

## The regulatory interaction of HSF1 with chaperones or chaperonins

In addition to the intrinsic regulatory mechanism, the interaction between HSF1 and other proteins, such as chaperones or chaperonins, is also prominent and considered to be a major regulatory mechanism at the macromolecular level (**Figure 2**).

### HSP90

HSP90 is one of the chaperones that interact with various client proteins. Once the nascent polypeptides or misfolded proteins are formed, they will be associated with the HSP70/HSP40/ADP complex to avoid accidental aggregation. HSP90 will then initiate the refolding function via a sequential process known as the HSP90 chaperone cycle that involves some partner proteins, immunophilin and ATP. After the cycle is completed, the original client proteins are transformed into mature and functional proteins 41.

Under normal or non-stressed conditions, HSF1 is associated with HSP90 that suppresses HSF1 trimer formation and promotes the monomeric/inactive form 42. The noncanonical regulation between HSP90 and HSF1 is related to the ATP-dependent conformational change of HSP90. When ATP binds to the N-terminus of HSP90, the ''closed'' conformation with transient dimerization is induced, which leads to an association between HSP90 and HSF1. Thus, HSP90 inhibitors, which bind to the N-terminal ATP-binding site of HSP90, competitively hinder the interaction between HSF1 and HSP90, thus prolonging the duration of HSF1 transcriptional activity 43.

### HSP70 and HSP40

The HSP70-HSP40 heterodimer participates in part of the protein folding process. HSP40 serves as a co-chaperone and assists HSP70 by bringing substrates to the peptide binding site on HSP70, stimulating ATP hydrolysis and changing the conformation of HSP70 to stabilize client interaction. Then, HSP40 dissociates from the HSP70/HSP40/ATP complex and a nucleotide-exchange factor (NEF) will attach to the nucleotide-binding domain of ADP-HSP70, triggering the exchange from ADP to ATP and completing the protein refolding process. Thus, conformational dynamics among the HSP70 nucleotide-binding domain, the HSP70 substrate-binding domain and the HSP40-HSP70 complex would affect the mechanism of HSP70 during folding 44, 45.

HSP70 possesses the suppressive function on HSR as well. Once the HSF1 trimer binds to the DNA and activates transcription, HSP70, in association with DnaJB1, binds to the transactivation domain of HSF1 to attenuate transcriptional activity. Further, HSP70 with DnaJB1 binds to the HR-B proximal binding site to dissemble the HSF1 trimer from the HSE and form an inert monomeric HSF1. The mechanism of HSF1 transcriptional attenuation and trimer disassembly will be held back under the extreme proteotoxic conditions due to the refolding process which requires HSP70 46. Since HSP70 overexpression facilitates downregulation of HSF1 DNA-binding activity, HSP70 is considered to be a negative regulator of HSF1 47.

### TRiC/CCT

Another category of proteins to regulate the HSF1 are chaperonins, the ATP-dependent molecular chaperones that form barrel-shaped complexes. Of these, the most notable is the T-complex protein Ring Complex (TRiC), also known as Chaperonin Containing TCP-1 (CCT). TRiC/CCT consists of two stacked anti-parallel rings of eight hetero-oligomers and forms a hexadecamer. Each subunit possesses its own function and CCT5 is thought to play a master role in assembly and disassembly of the complex 48.

Like HSP90 and HSP70-HSP40, TRiC/CCT interacts with and represses HSF1 34. TRiC/CCT also interacts with other chaperones to modulate proteostasis. For example, the cooperation of TRiC/CCT and prefoldin, an ATP-independent chaperone, contributes to the successful folding process, and disrupting the electrostatic interaction between each leads to substrate aggregation 49.

## Post translational modifications (PTMs)

Covalent modifications of HSF1 on amino acid residues are considered to be an influential process to affect different physiological states, including protein stability, oligomerization, transcriptional activity, and so on 22. Intriguingly, various PTMs, such as phosphorylation, acetylation and SUMOylation, that occur mainly on amino residues encoded by the regulatory domain, may cause either inhibitory or stimulatory effects on the physiological and pathological state of HSF1 22, 31.

### Phosphorylation

Among the PTMs, phosphorylation of serine or threonine residues is the most prevalent and different phosphorylation sites mediated via multiple regulatory enzymes may result in various effects (**Table 1** and **Table 2**). Among these phosphorylation sites, serine 326 residue plays a critical role in HSF1 induction 50. Phosphorylation of HSF1 at serine 326 (HSF1 pSer326) stimulates HSR and is related to the poor prognosis of cancer. HSF1 pSer326 leads to HSF1 activation and the induction of HSP27 expression in cancer cells with higher expression of stem cell-related genes, negatively impacting overall survival in patients 51. Previous studies on multiple myeloma indicated that the development of proteasome inhibitor resistance (e.g., bortezomib), is attributed to HSF1 pSer326, which upregulates HSR and attenuates apoptosis 52. Furthermore, bortezomib treatment is beneficial in pediatric acute myeloid leukemia patients with low HSF1 pSer326 as compared to those with high levels of HSF1 pSer326 53. In order to tackle resistance development, specific multi-kinase inhibitors are under development to overcome resistance due to proteasome inhibitor induced HSF1 pSer326 and may serve as a novel means to treat cancer relapse 54.

HSF1 was also found to impact gene expression epigenetically. The PLK-mediated phosphorylation of HSF1 pSer419 recruits the TRRAP-TIP60 complex, a histone acetyltransferase, to the HSP72 promoter to thereby upregulate HSP72 expression. Such epigenetic regulation may also involve in tripartite motif (TRIM) 33 and TRIM24 as both acetylated histone reader and ubiquitin writer to initiate an active chromatin state and enhance transcriptional activity through RNA polymerase II. Interestingly, HSF1 pSer419 is related to the proliferation of melanoma cells, promoting tumor formation. Therefore, this unique pathway inclusive of HSF1 phosphorylation and the TRRAP-TIP60 complex might also be a promising target in the treatment of melanoma 77.

Conversely, phosphorylation of HSF1, such as residues Ser303 and Ser307, would repress its transcriptional activity. Cyclosporin A, a traditional non-cytotoxic immunosuppressant, enhances the phosphorylation of Ser303 and Ser307 on HSF1 mediated by ERK1/2, GSK3β and CK2 to promote cell death and overcome chemotherapy resistance 63. On the other hand, increased levels of HSF1 pSer303 and HSF1 pSer307, induced by the angiotensin II in hypertension patients, will result in cardiac hypertrophy and cardiomyocyte apoptosis, which leads to the exacerbation of heart failure 64.

### Acetylation

Acetylation of lysine residues can have numerous effects on protein function and stability. The most prominent residue for HSF1 acetylation is K80, located in the DNA binding domain. The acetylation of K80 has an inhibitory effect on DNA binding that abrogates HSF1 transcriptional activity. Two main regulators involved in this pathway are p300 and SIRT1, mediating acetylation and deacetylation, respectively 80, 81.

The acetyltransferase p300 not only attenuates HSR by acetylating K80 and K118 but also stabilizes HSF1 to prevent proteasomal degradation by acetylating K208 and K298 80. The protective effect of methylene blue (MB) from oxidative stress induced cellular dysfunction is mediated by an increase in the expression of HSF1 following the formation of the HSF1/p300 interaction complex 82.

Another important regulator is SIRT1, a nicotinamide adenosine dinucleotide-dependent deacetylase. Overexpression of SIRT1 prevents HSF1 acetylation, thereby promoting binding of HSF1 to HSE and the onset of the HSR 81. Because upregulation of HSP is SIRT1-dependent, treatment with salvianolic acid A could increase SIRT activation and relieve liver fibrosis due to the inhibition of endoplasmic reticulum stress 83. Further, modulators of SIRT1 have been found to affect HSF1 and may serve as innovative therapeutic agents to regulate the HSR 84.

### SUMOylation

Small ubiquitin-related modifier (SUMO), which targets the SUMO acceptor residue, usually lysine, mainly causes a repressive effect of the target protein 85. In contrast to other modifications, SUMOylation depends on phosphorylation on the proximate serine on HSF1, which is known as phosphorylation-dependent SUMOylation. The motif ΨKxExxSP, in which Ψ stands for the large hydrophobic residue and x stands for any amino acid, is identified as the SUMOylation consensus site, especially on HSF1 Lys298 and Ser303 86. Recently, it was shown *in vitro* that heat inducible SUMOylation is magnified by phosphorylation not only at Ser303 but also at Ser307. Further, SUMOylation promotes an inhibitory effect followed by HSP70 activation to restore the HSF1 inert state and attenuate the HSR 87.

## Non-coding RNA

In addition to protein-protein interactions and PTMs, non-coding RNA (ncRNA), defined as an RNA without the ability to translate into protein, also plays an important role in the regulation of HSF1. For instance, some ncRNAs suppress mRNA transcription followed by the onset of the HSR. The most prominent one is *Alu* 88. As small interspersed nuclear elements, *Alu* could bind to RNA polymerase II directly, disrupting contacts with the promoter and suppressing basal transcription 88, 89. Intriguingly, *Alu* processing was accelerated by HSF1 and an increase of *Alu* processing was found in patients with Alzheimer's disease, suggesting that HSF1-*Alu* mediated gene expression plays an important role in Alzheimer's disease pathogenesis 90.

HSF1-associated ncRNAs were found in different types of cancers. In hepatocellular carcinoma cells (HCCs) for example, microRNA-644a (miR-644a) downregulates HSF1 by binding to its 3'-untranslated region, promoting an inhibition of proliferation and the onset of apoptosis. Moreover, the level of miR-644a in HCCs is suppressed, which indicates that miR-644a might be a prognostic biomarker or potential target to treat HCCs 91. On the other hand, a long non-coding RNA, lncRNA inducing MHC-I and immunogenicity of tumor (LIMIT), is related to immunotherapy. LIMIT *cis*-activates the proximate gene cluster guanylate binding proteins (GBPs). Once the GBPs are overexpressed, they disrupt the HSP90-HSF1 complex, releasing and activating HSF1 to stimulate MHC-I expression and boost antitumor immunity 92. Intriguingly, the miR-644a-HSF1 axis triggers the onset of the apoptotic pathway whereas the LIMIT-GBP-HSF1 axis impacts immunotherapy - both attenuating tumor progression by suppression or stimulation of HSF1, respectively. Although the concepts are completely different, they manifest similar outcomes to attenuate cancer.

## HSF1 regulates several cancer-related cellular functions

**Figure 3:**
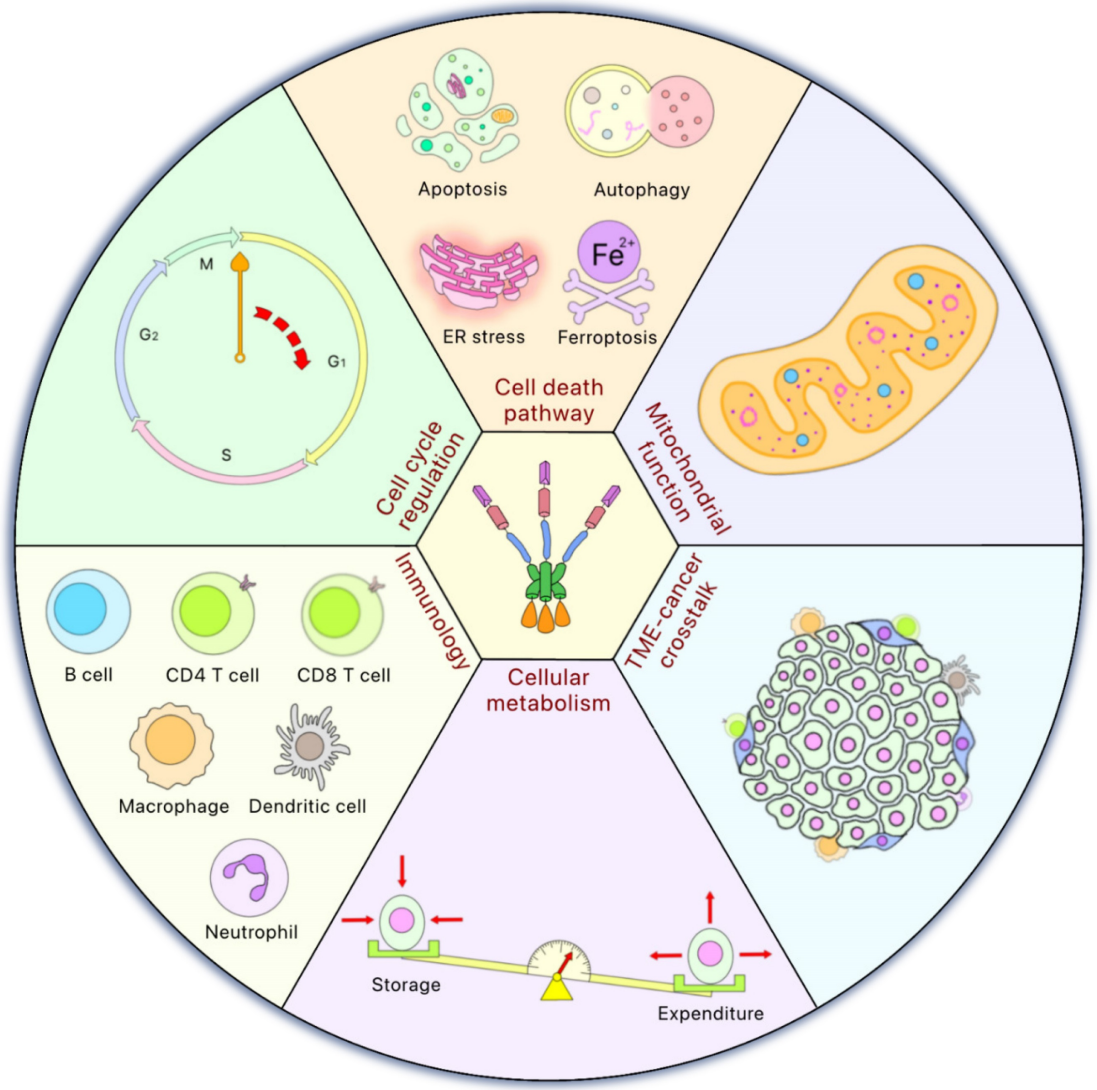
** HSF1 is involved in multiple cellular functions**. HSF1 is involved in various cellular functions to maintain normal physiological status. Moreover, some of these functions of HSF1 may also play an essential role in cancer development such as cell cycle regulation to control cell proliferation, cell death pathway activation, mitochondrial function, important to cope with the oxidative stress, tumor microenvironment (TME)-cancer crosstalk to promote malignancy, cellular metabolism related to the equilibrium between energy storage and expenditure, and immunology which is now mainstream for cancer therapy.

Although HSF1 plays a critical role in proteostasis, HSF1 is also a “multitasker” that influences many cellular functions. Mounting evidence has suggested that HSF1 plays multifaceted roles in various cellular responses associated with tumorigenesis, such as the alteration of the tumor microenvironment, genome repair, among other critical cellular response pathways 93. HSF1 also plays roles in cell death pathways, including apoptosis 94, autophagy 95 and ferroptosis 96. In general, cancer cells express higher levels of HSF1 as compared to normal, non-transformed cells 93, 97 and HSF1 activation in the stroma indicates poor prognosis and an undesirable outcome 98 (**Figure 3**).

HSF1 is related to the HSP-dependent pathway as well as other oncogenic pathways, such as the RAS-MAPK or PI3K-AKT-mTOR pathways that transduce signals regulated by HSF1 and promote tumorigenesis 99. Furthermore, many proteins associated with tumor cell survival are HSP90 client proteins, and tumor cells demonstrate greater dependence on HSP90 100. Therefore, HSF1 inhibitors could be developed as a therapeutic agent to antagonize malignant progression 20.

### HSF1 in cell cycle regulation and cell proliferation

Since HSF1 is induced and binds to HSE during the G₁ phase, in the absence of stress, this implies a role for HSF1 in cell cycle progression 101. In mouse embryonic fibroblasts, a G₂ cell cycle arrest was observed in HSF1 expressing cells to protect against apoptosis induced by irradiation or cisplatin treatment 102. A transient G₂/M arrest was also observed after conditioning and lethal heat 103. Moreover, resistance to radiation-induced G₂ arrest and double-stranded DNA breaks repair is seen upon decreased expression of HSF1 104. Thus, HSF1-mediated regulation of the cell cycle could be a mechanism of drug resistance and treatment failure. A mechanism of HSF1-mediated cell cycle regulation has been identified. PLK1, one of the major mitosis-regulating kinases, phosphorylates HSF1 (Ser216) and promotes an interaction with CDC20 to activate the spindle checkpoint. Then, HSF1 is degraded following ubiquitin modification, CDC20 is released and in-turn binds to the anaphase-promoting complex (APC), allowing the transition of metaphase to anaphase 60. To sum up, HSF1 serves as a regulator of mitosis and is essential for mitotic progression, an important mechanism related to cancer therapy.

HSF1 is also involved in pathways that regulate cell proliferation, such as the mammalian target of rapamycin (mTOR), mitogen-activated protein kinase (MAPK) or epidermal growth factor receptor (EGFR) pathways. HSF1 stimulates mTOR activation, promoting carcinogenesis. Intriguingly, this may depend on glutaminolysis via the upregulation of glutaminase-1 in colorectal cancer 105. HSF1 could impact AKT as well. In triple negative breast cancer, the transcription factor YY1 activates the expression of HSF1 that triggers AKT signaling to promote cell proliferation 106. Of note, two distinct mTOR complexes, mTORC1 and mTORC2, possess different paths to regulate HSF1. HSF1 plays a chief role in sustaining mTORC1 activity by suppressing c-JUN N-terminal kinase (JNK), which leads to the positive regulation of cell, organ, and body sizes 107. On the other hand, mTORC2 regulation is related to one of its subunits, RICTOR. The resulting AKT-HSF1-HuR pathway has been observed in glioblastoma multiforme and identified as a feed-forward loop mechanism to regulate RICTOR expression and mTORC2 activity. Knockdown of each of these factors decreases migration and invasion capacity 72. Moreover, AKT, a positive regulator of mTOR, also impacts HSF1 since specific inhibitors of AKT or mTOR inhibit HSF1 phosphorylation in PI3K-AKT-mTOR-constitutively activated cells 108. Thus, the HSF1-AKT-mTOR axis exerts multiple mechanisms affecting cell cycle and proliferation.

Proteins of the MAPK pathway controlling cell proliferation have been implicated in carcinogenesis and are found to functionally interact with HSF1. For example, fucosyltransferase IV (FUT4) promotes cell proliferation via both MAPK and PI3K-AKT pathways and was found to be upregulated by HSF1 to promote cancer cell proliferation 109. Notably, p38 MAPK phosphorylates HSF1 at Ser326 and elevated levels of pSer326 phosphorylation results in activation of HSF1 67.

HSF1 is also involved in the activation of HER2, an EGFR family member, which plays a critical role in breast cancer. In HER2 expressing cells, downregulation of HSF1 contributes to the decreased proliferation and senescence 110. Simultaneously, HER2-overexpressing cancer cells activate HSF1 via the PI3K-AKT axis by phosphorylation of HSF1 at Ser326 followed by the stabilization of tumor-promoting proteins perceived as HSP90 clients 111. Our lab has shown that HER2 upregulates HSF1 synthesis, resulting in transcriptional activation of LDHA and glycolysis. Such dysregulated glucose metabolism leads to breast cancer cells that present with resistance to trastuzumab, a HER2 targeting drug 7, 112. Consistent with these findings, resistance to lapatinib, another HER2 inhibitor, also was found to implicate HSF1 113. Accordingly, HSF1 inhibitors might help decrease resistance in HER2 targeted therapy paradigms.

### HSF1 in regulating cell death pathways

HSF1 regulates several cell death pathways, including apoptosis, autophagy, endoplasmic reticulum (ER) stress and ferroptosis, all critical for cancer cell survival.

Apoptosis is a central programmed cell death mechanism and HSF1 plays an important role in suppressing this pathway, likely mediated via the tumor suppressor gene *p53* 94. Additionally, HSF1 is related to doxorubicin-induced cardiotoxicity. HSF1 promotes HSP27 expression and once HSP27 is phosphorylated by p38 MAPK, *p53* binds to phosphorylated-HSP27 and increases p21 transcription, which leads to enhanced cell survival and attenuation of cardiotoxicity 114. This has also been observed for the IGFIIR-mediated apoptotic pathway. Doxorubicin blocks the CHIP-HSF1 interaction, leading to HSF1 degradation, and the upregulation and translocation of IGFIIR to induce caspase-3 activation. This ultimately leads to enhanced cardiomyocyte apoptosis 115. Therefore, HSF1 activation may reverse doxorubicin-induced cardiotoxicity although doxorubicin-resistance in HSF1 overexpression cancer cells has also been reported 116. Moreover, HSF1 expression correlates with upregulation of Bcl-2-associated athanogene-3 (BAG3), an HSP70 co-chaperone. HSF1 stimulates the expression of both HSP70 and BAG3, which stabilizes the anti-apoptotic Bcl-2 proteins and leads to the inhibition of apoptosis 117. Notably, this pathway is suggested to account for the observed cell death resistance in glioma 118, as well as the reduced sensitivity of specific drugs in gastric cancer treatment 119.

Another pathway to maintain cellular homeostasis is autophagy. Malfunction of autophagy leads to various human pathological conditions including cancer 120. HSF1 was found to regulate sequestosome-1 (SQSTM1), a prominent autophagic receptor that regulates inclusion body formation and the degradation of aggregated proteins. Upon HSF1 activation, the phosphorylation of SQSTM1 is induced by mTORC1, CSNK1 and TBK1. This enhances the autophagic clearance of these harmful, aggregated proteins 121. Further, chemoresistance is also associated with HSF1 enhanced autophagy. We have shown that upon carboplatin treatment, HSF1 is activated and transcriptionally upregulates autophagy-related gene 7 (ATG7), which triggers autophagy and increases cell survival and chemoresistance 10. A similar situation was found in oxaliplatin-treated colorectal cancer. HSF1 causes miR-135b-5p overexpression which prevents unc-51-like kinase 1 (ULK1), a chief autophagy regulator, from ubiquitination by mitochondrial ubiquitin ligase 1 (MUL1) which results in the initiation of autophagy 122.

The unfolded protein response (UPR) is a cell survival mechanism by which cells can relieve the stress of misfolded protein accumulation in the endoplasmic reticulum (ER), a stress induced by a wide variety of pathophysiologic and pharmacologic factors. Failure to alleviate ER stress may result in apoptosis 123. Therefore, HSF1 could relieve ER stress 124 and its capacity to prevent ER stress-mediated apoptosis 125.

Last but not least is ferroptosis, a novel cell death pathway driven by iron-dependent phospholipid peroxidation. Ferroptosis is repressed by glutathione peroxidase 4 (GPX4), and is involved in drug sensitivity and resistance to cancer cells, considered to be a promising target for novel anticancer agents 126. Although there is no direct regulatory relationship reported so far, HSF1 has been implicated in the ferroptosis pathway. Intriguingly, heat shock protein beta-1 (HSPB1, also known as HSP27), a direct transcriptional target gene of HSF1, is upregulated by erastin-induced ferroptosis *in vitro*. Additionally, knockdown of either HSF1 or HSPB1 boosts the anticancer activity of erastin *in vivo*. Thus, it appears that ferroptosis is regulated negatively by the HSF1-HSPB1 pathway 96. Furthermore, a recent report showed that 4-hydroxynonenal, a lipid peroxidation metabolite, stimulates HSF1 phosphorylation and activation via p38 MAPK pathway, which leads to the activation of prominin-2 transcription and promotes the ferroptosis resistance 127. Further investigation of the role of HSF1 in ferroptosis may lead to better understanding of this new pathway of cell death and reveal new opportunities for HSF1-targeted drug development.

### HSF1 in oxidative stress and mitochondrial function

Heat shock produces another type of stress in the form of reactive oxygen species (ROS). Recently, it was identified that ROS serves as a key factor to induce ferroptosis 126. Interestingly, ROS also triggers the p38 MAPK pathway, the upstream regulator of HSF1, as well as the overexpression of two survival markers, such as HSP70 to maintain proteostasis and manganese superoxide dismutase to counteract oxidative stress 128. Thus, ROS might manifest both ferroptosis meditated cell death as well as survival signals that lead to an equilibration between cell death and survival outcomes. Moreover, HSF2, a homolog of HSF1, functions in cooperation with HSF1 as the heterotrimer that binds to oxidative stress-specific target genes in response to increased ROS-mediated oxidative stress 129. Given the recent findings that the interaction between HSF1 and HSF2 is essential for cancer gene expression 24, 129, an important role of HSF2 in cancer development may become increasingly significant.

The mitochondrion is an essential energy-producing organelle and plays critical roles in a large range of cellular functions. HSF1 maintains mitochondrial function in response to mitochondrial proteotoxic stress, which is attributed to the upregulation of mitochondrial chaperone expression via HSF1, such as HSP60, HSP10 and mitochondrial HSP70 130. This protective mechanism also involves single-strand DNA-binding protein 1 (SSBP1) which is related to the replication and maintenance of mitochondrial DNA. It has been found that the HSF1-SSBP1 complex supports both cell survival and mitochondrial function. Thus, they are indispensable due to their regulation of mitochondrial chaperones 130, 131.

In addition, the downregulation of the second mitochondria-derived activator of caspase (SMAC) leads to decreased apoptosis and drastically increased cell proliferation in pancreatic cancer cells. HSF1 manifests an inhibitory effect on mitochondrial apoptosis by down-regulating SMAC expression, suggested to correlate with poor prognosis 132. Intriguingly, HSR promotes perinuclear mitochondrial clustering that boosts ROS levels in the nucleus and leads to the activation of hypoxia-inducible factor-1α (HIF-1α), required for HSF1 activation during HSR 133.

### HSF1 in tumor microenvironment (TME)

The tumor microenvironment (TME) consists of multiple non-malignant cells in proximity with the tumor and involves the interplay between cancer cells and the cells and factors of the TME to support malignancy. Within the TME, stromal HSF1 activation drives a transcriptional program to promote malignant phenotypes and is related to poor outcome observed in breast cancer and lung cancer patients 98. Similarly, high HSF1 expression in stromal cells reflects poor prognosis in esophageal squamous cell carcinoma patients 134, suggesting that HSF1 may be involved in TME regulation.

A major component of the TME is cancer-associated fibroblasts (CAFs) that are crucial in cancer progression. For example, two protumorigenic proteins, inhibin subunit beta A (INHBA) and thrombospondin 2 (TSP2), are secreted from CAFs to the TME via extracellular vehicles, found to be an HSF1-dependent mechanism that promotes gastric cancer. Likewise, HSF1 activation in gastric CAFs is associated with poor outcome 135. Another mechanism related to HSF1 has been identified in BRCA-mutated pancreatic ductal adenocarcinoma. HSF1 activation is induced in pancreatic stellate cells due to stress imposed on the TME mediated by BRCA-mutated cancer cells. These pancreatic stellate cells would be reprogrammed from myofibroblastic into immune-regulatory clusterin-positive CAFs which function as both T-cell immune suppressor cells and as altered extracellular matrix organization modulator cells 136. On the other hand, HSF1 in cancer cells could be induced by CAFs as well. In gallbladder cancer cells, integrin α2 on the surface is activated by thrombospondin 4 (TSP4) secreted from CAFs. This is followed by phosphorylation of AKT and HSF1, leading to cancer stem-like traits. HSF1 activation also triggers TGF-β paracrine signaling and the onset of transdifferentiation from reactive fibroblasts to CAFs, highlighting a positive feedback loop to drive the growth of gallbladder cancer cells 70. To sum up, due to the multiple roles of HSF1 found in various cancer types and the associated TME, crosstalk between the TME and cancer cells related to HSF1 may be a novel target to treat solid tumors in the future.

### HSF1 in cellular metabolism

HSF1 is also involved in cellular energy metabolism, reported to be dysregulated in HSF1-null mice and HSF1 activation increases energy expenditure 137. Moreover, HSF1 promotes the conversion of white fat to brown fat, inducing thermogenesis and protecting against obesity onset 137, 138.

HSF1 ablation affects various cellular metabolic processes such as suppressing mitochondria respiratory capacity. Intriguingly, HSF1 deletion inhibits the NAD^+^ and ATP regeneration process which is strongly related to the cellular energetic deficits seen when HSF1 expression is lost 139. A similar situation was observed in human acute T-cell lymphoblastic leukemia cells. HSF1 inhibition negatively regulates anabolism by downregulation of mTORC1. The antileukemic activity is then antagonized by the activation of adaptive glutamine metabolism following HSF1 inhibition 140. Further, HSF1 inhibition downregulates mevalonate and cholesterol biosynthesis-related gene expression in hepatocellular carcinoma. Once HSF1 is inhibited, simvastatin-induced cholesterol-depletion then promotes antiproliferative effects 141. Furthermore, cellular metabolism is associated with therapeutic resistance. Our research has demonstrated that oncogene HER2 (ErbB2)-upregulated HSF1 leads to enhanced glycolysis by transactivation of LDHA, a key enzyme in the glycolytic pathway, and the increase in glycolysis is related to cancer cell resistance to the HER2 targeting agent Herceptin 7, 112. Similarly, HSF1 stimulates the expression of pyruvate dehydrogenase kinase 3 (PDK3), which promotes glycolysis that supports cancer progression and chemoresistance in gastric cancer cells. Importantly, inhibition of PDK3 can reverse this observed chemoresistance. Mechanistically, PDK3 interacts with HSF1 to prevent HSF1 from FBXW7-dependent polyubiquitination and degradation 142. Thus, blockade of the HSF1-PDK3 positive feedback loop may help to overcome chemoresistance.

### HSF1 in immunology

Generally, inflammatory signals promote tumorigenesis, a process that is suggested to be regulated by HSF1. HSF1 is activated via JNK2-mediated phosphorylation to then demethylate the IL-6 promoter, enhancing IL-6 expression. Further, IL-6 signaling suppresses miR-200c, which leads to enhanced JNK2 activation. This constitutively active inflammatory pathway could be found in human cancer cells 143. As for the proinflammatory signal, HSF1 boosts TNF-α induction and promotes NF-κB signaling pathway activation through the TNF-α receptor 1 (TNFR1). Of note, TNFR1-knockdown in colon cancer cells show less cell proliferation and migration/invasion potential than control cells following heat shock. Thus, HSF1, as an upstream regulator, is considered to be a target and its inhibition might control inflammation and related diseases such as cancer 144.

In diverse cancers, HSF1 upregulation correlates with the expression of immune checkpoint proteins, which might contribute to poor prognosis and immunotherapy resistance 97. The phosphorylation of HSF1 (threonine 120) by PIM2, an oncogenic serine/threonine kinase, plays a crucial role in up-regulating the expression of PD-L1 in breast cancer cells. Once HSF1 is phosphorylated, the elevated levels of PD-L1 will then exacerbate immune escape, leading to cancer cell proliferation 55. Functional PD-L1 upregulation was also found in oral squamous cell carcinoma when HSF1 pSer326 is induced by cisplatin. It has also been found that PD-L1 is a client protein of HSP90 145. Thus, the HSF1-HSP90 axis might be required to overcome immunotherapy resistance.

HSF1 has also been found to promote antitumor effects. The lncRNA LIMIT enhances immunotherapy efficacy in response to IFNγ. Intriguingly, this enhancement of immunotherapy by LIMIT may be at least in part attributed to activation of HSF1, found to stimulate MHC-I expression and antitumor immunity 92. Further, HSF1 knockdown leads to downregulation of major histocompatibility class I chain-related proteins B (MICB), a natural killer cell‑activating ligand on tumor cells, and decreased NK cell cytotoxicity 146. Therefore, HSF1 might be a multifunctional molecule to influence the antitumor immune response. Future studies are warranted to determine the precise function for HSF1 with regard to immunotherapy.

HSF1 is virtually always overexpressed in human malignancies. HSF1 is one of the pan-cancer targets because of its upregulation and significance for the maintenance of numerous oncogenic pathways. Select HSR pathway inhibitors have produced encouraging effects in preclinical models and smaller-scale clinical investigations 147.

The modification of immunosuppressive activity by malignancies is a novel approach for investigation of anti-tumor effects of HSR inhibition, which has been unable to be detected by conventional models such as using immunodeficient mice.

Immune checkpoint inhibitors and other currently utilized anti-cancer medications may be more effective when used in combination with HSR-targeting medicines. To successfully target HSR components, particularly HSF1, for cancer therapy, it is necessary to design more focused inhibitors with improved pharmacokinetic features.

In order to pinpoint individual HSR proteins as therapeutically promising targets for drug development, the initial screening process usually involves a strong correlation with poor prognosis or selective overexpression in malignancy. Identification of HSR components that display a pronounced pattern of co-expression and/or reliance for an oncogene, that by itself is not very druggable, is a different strategy that should be considered for novel drug targets.

## Clinical relevance of HSF1 in different cancer types

HSF1 is recognized as a potent and multifaceted modifier of the cell stress response and demonstrated to regulate many functions related to cancer cell malignancy, i.e., modifying the tumor microenvironment, maintaining protein homeostasis, reprogramming metabolism, promoting proliferation and migration, repairing the genome, and inhibiting apoptosis 93. **Table 3** lists a few representative examples of the roles of HSF1 in cancer development.

## HSF1 Inhibitors

Targeting HSF1 for cancer therapy is still in the pre-clinical stage but it has been suggested to be a promising modality in cancer treatment. For example, loss of HSF1 activity suppressed aneuploidy and proliferation of cancer cells in many cancer types 18.

Despite the successful inhibition of HSF1 reported in *in vitro* experiments and in animal models, each inhibitor has limitations in clinical use. HSF1 plays an important role in both cancer cells and normal cells under stress. HSF1 inhibition, which was originally intended to be an anti-cancer modality, should not be toxic to normal cells. As a result, it is critical for its use in cancer treatment to specifically identify and target cancer cells in order to minimize cytotoxic effects on normal cells. This necessitates the refinement of existing compounds, for example, via synthetic approaches that alter functional groups/motifs, or the discovery and isolation of new natural products capable of overcoming potential off target issues 168.

Developing HSF1 inhibitor is difficult due to a lack of potential target sites in its tertiary structure. HSF-1 is a ligand-less transcription factor with poor 'druggability' traits 168, 169. Furthermore, its complicated activation process involves multiple factors such as a multichaperone complex and numerous post translational modifications. Nevertheless, potential HSF1 inhibitors have been developed, often derived from natural products or synthetic chemical structures (**Table 4**). Each hit compound may have different mechanisms of action for such inhibitory effects of HSF1, including interference with HSP90-HSF1 dissociation or HSF1 translocation and trimerization. Other mechanisms involve inhibition of HSF1 by targeting post translational modifications or intramolecular interactions with multichaperone complexes or cellular proteins 170, 171. Unfortunately, in many cases, the exact mechanism of action and drug specificity of these inhibitors is still unclear.

HSF1 is a protein that regulates the expression of other proteins involved in cellular stress response, such as HSPs. KRIBB11 is a compound that can inhibit the activity of HSF1 by preventing a protein called positive transcription elongation factor b (P-TEFb) from binding to the promoter region of HSP70 gene. This impairs the ability of HSF1 to activate the transcription of HSP70 and other stress response genes 20, 172. Animal models administered KRIBB11 showed HSF1 inhibitory activity, which was characterized by decreased HSP70 expression and decreased tumor mass volume 172. Application of KRIBB11 significantly inhibited lymphatic metastasis of bladder cancer with no significant toxicity 173. Moreover, the combination of KRIBB11 and an Aurora Kinase A (AURKA) inhibitor (danusertib) showed excellent anti-tumor effects on liver cancer *in vitro* and *in vivo*. Importantly, this combination induces cancer cell apoptosis and attenuates tumor growth by activating the ER stress response 174. However, studies on KRIBB11 as an HSF1 inhibitor have shown some inconsistent results. For example, in a study related to PROM2 and ferroptosis, treatment with KRIBB11 alone had no significant effect on the expression, phosphorylation and nuclear localization of HSF1 127, indicating it does not directly inhibit HSF1.

Recently, deoxyschizandrin / schizandrin A (Sch-A), a bioactive molecule derived from a plant species well known in Far Eastern medicine (*Schisandra chinensis*), was shown to inhibit HSF1. Sch-A binds to HSF1 directly and inducing conformational changes to the binding site, which causes cell cycle arrest and cell death in human colorectal cancer cells. In addition, Sch-A suppressed heat shock-induced HSR activity and downregulated mRNA transcription and protein expression of HSF1 downstream genes, such as HSP70, HSP27 and HSP90, under both heat shock and basal conditions 175, 176.

Quercetin, a plant pigment of the flavonoid family, is one of the most prominent dietary antioxidants. It is present in a variety of foods, including onions, tea, wine, apples, and berries, and is responsible for various health benefits. Quercetin has been shown to prevent HSF1 binding to HSEs on known HSF1 target genes 177. Additionally, quercetin also suppressed HSP70 accumulation in tumors after combination therapy and promoted cell apoptosis via the HSF1 pathway. In an animal study, the combination of quercetin and radiofrequency ablation (RFA) activates caspase 3, which leads to apoptosis and decreases HSP70 expression 178.

Triptolide, an active ingredient in *Tripterygium wilfordii*, was found to be an HSF1 inhibitor. HSF1 is overexpressed in chronic lymphocytic leukemia (CLL) and treatment with triptolide induces apoptosis in cultured and primary CLL B-cells. Knockdown of HSF1 or its inhibition with triptolide results in the reduction of HSP90 and disrupts the cytosolic complex between HSF1, p97 (a segregase with ATPase activity), HSP90 and the HSP90 deacetylase-Histone deacetylase 6 (HDAC6). Consequently, HSF1 inhibition results in HSP90 acetylation and abrogation of its chaperone function leading to suppression of malignancy 179.

Triazole nucleoside exhibits potent anticancer activity in prostate cancer 180. This agent downregulates HSF1 and multiple types of chaperones consisting of HSP27, HSP70 and HSP90. Additionally, this compound inhibits AR expression and arrests the cell cycle in prostate cancer cells 180.

PGPIPN, a hexapeptide derived from the 63-68 amino acid residue of bovine β-casein 181, reduced the resistance of ovarian cancer cells to cisplatin by affecting the HSF1/HSP70 signaling pathway. Specifically, PGPIPN affected the expression levels of HSF1, HSP70 and MDR1 genes. Of note, PGPIPN can enhance the sensitivity of cancer cells to cisplatin, suggesting that PGPIPN and cisplatin may act synergistically when treating ovarian cancer 182.

Recently, a synthetic compound, Direct Targeted HSF1 Inhibitor (DTHIB, also known as SISU-102), was identified by screening for interactions between the structurally well-ordered HSF1 DBD and small molecules. DTHIB selectively engaged with HSF1 and inhibited core HSF1 functions by accelerating the rate of active, trimeric, nuclear HSF1 degradation and therefore potently inhibiting HSF1 and coordinately reducing abundance of the multichaperone complex. In prostate cancer, compared to enzalutamide, an anti-androgen receptor agent, DTHIB shows superiority in decreasing cell viability and inhibiting AR signaling and PSA expression. Furthermore, DTHIB also inhibits cell proliferation and reduces tumor growth. In mouse models, DTHIB showed no obvious side effects such as weight loss or behavioral changes 164. More recently, DTHIB has been shown, in an acute myeloid leukemia (AML) animal model, to specifically suppress leukemia stem cell self-renewal while sparing normal hematopoietic stem/progenitor cells 183. In this study, DTHIB inhibited the expression of HSF1 targets, HSP90 and suppresses mitochondrial oxidative phosphorylation by downregulation of SDHC, a key enzyme complex of the tricarboxylic acid cycle 183.

A recent discovery using the chemical probe bisamide, CCT251236, showed impressive results for HSF1 inhibition and antitumor efficacy in a preclinical model using ovarian cancer cells 169. In addition, in a study using multiple myeloma cells, an optimized derivative, CCT361814, was shown to inhibit the heat shock pathway, as indicated by a concentration-dependent loss of HSP27 and HSP72 protein levels and potent anti-myeloma activity 184. Currently, CCT361814, also known as NXP800, is entering a phase I clinical trial for advanced solid tumors 185. With these promising pre-clinical results, we look forward to learning the outcome of the clinical study results.

## Conclusion

HSF1 plays an essential role in cells by functioning as a master regulator of the cellular stress response. In addition to the heat shock response that protects cells from the damage induced by proteotoxic stress, HSF1 also regulates a non-heat shock responsive network to handle metabolic, chemical, and genetic stress. A large amount of evidence supports an important role for HSF1 in cancer development, during which HSF1 modulates multiple pathways including cell proliferation, cell survival, protein synthesis and energy metabolism. Not surprisingly, new molecular mechanisms underlying the functions of HSF1 have been continuously discovered, which provide strong evidence to support the notion that HSF1 may serve as an excellent target for novel cancer treatment strategies.

Because of its “undruggable” structural properties, the development of a small molecule inhibitor of HSF1 is difficult. Most current HSF1 inhibitors are indirectly inhibiting HSF1 functions instead of directly targeting HSF1 itself, which could explain why they lack specificity and potency. Even those that are reported to be directed are tested at high micromolar concentrations (often >100 μM) and caution is warranted. Another consideration is that given the important cellular functions of HSF1, its inhibition may cause toxic effects to normal tissues. However, because HSF1 is a ligand-less transcription factor, standard drug discovery strategies and direct inhibition with small molecules are unlikely to be effective. Thus, through cell-based phenotypic screening, inhibitors of HSF1-mediated transcription that antagonizes the HSF1 pathway, without necessarily binding directly to HSF1, may be feasible.

Recently reported direct HSF1 inhibitors such as DTHIB and CCT361814 have brought new hope to HSF1 drug development. These two inhibitors appear to have high potency and high specificity for the suppression of tumor growth, but low toxicity to normal tissues in pre-clinical animal studies. Promisingly, CCT361814 has entered a Phase I clinical trial 185, 199. It is anticipated that new generations of HSF1 inhibitors, especially those directly targeting HSF1 itself, should be developed. Moreover, future studies should be performed to identify biomarkers for patient selection and therapeutic effect monitoring. It is hopeful that we would be able to develop an HSF1 targeting therapy which shows therapeutic benefits in patients in the near future.

## Figures and Tables

**Table 1:** List of HSF1 phosphorylation sites, involved enzymes and effects

Phosphorylation site	Enzymes	Effects	Reference
Thr120	PIM2	Induce PD-L1 expression	55
Ser121	MK2	Enhance HSF1 binding to HSP90 and inactivate HSF1	56
	AMPK	Inactivate HSF1	57
Thr142	AKT1	Enhance trimerization of HSF1	58
	CK2	Enhance trimerization of HSF1 and activate HSF1	59
Ser216	PLK1	Regulate mitotic progression	60
Ser230	AKT1	Enhance HSF1 association with general transcriptional factors	58
	AKT, DAPK	Activate HSF1	61, 62
Ser303	ERK, GSK3 and CK2	Repress HSF1 and enhance cell death	63-65
	AMPKα	Regulate HSPs negatively	66
	p38 MAPK	Repress HSF1	67
Ser307	ERK, GSK3 and CK2	Repress HSF1 and enhance cell death	63, 64
	MAPK, p38 MAPK	Repress HSF1	65, 67
Ser320	DYRK2, PKAcα	Activate HSF1	68, 69
Ser326	AKT, ERK1/2, CDK8, MEK, mTORC1, DYRK2, p38 MAPK	Activate HSF1 and promote cell proliferation	67, 68, 70-75
	AKT1	Enhance HSF1 association with general transcriptional factors	58
Ser333	PKCθ	Activate HSF1	76
Ser363	PKCα, PKCζ	Repress HSF1	65
Ser419	PLK1	Promote cell proliferation	77
Thr527	AKT1	Enhance HSF1 association with general transcriptional factors	58

**Table 2:** List of HSF1 dephosphorylation sites, involved enzymes and effects

Dephosphorylation site	Enzymes	Effects	Reference
Ser121	PP2A	Activate HSF1 and promote cell proliferation	78
Ser307	PP2A	Activate HSF1 and promote cell proliferation	78
Ser314	PP2A	Activate HSF1 and promote cell proliferation	78
Thr323	PP2A	Activate HSF1 and promote cell proliferation	78
Ser363	PP2A	Induce HSF1 target genes	79
Thr367	PP2A	Activate HSF1 and promote cell proliferation	78

**Table 3:** Summary of HSF1 expression and function in different types of cancer

Cancer Type	Effect on tumorigenesis	Mechanism	Reference
Breast Cancer	Promotes glycolysis, cell growth and invasion	HSF1/LDHA axis	7, 148
	Mitogenic action, motility and cell spreading	ERα	149, 150
	Apoptosis	MicroRNA-615-5p	151, 152
	Proliferation and migration	FAM3C-YY1-HSF1 axis	153-156
Lung Cancer	Apoptosis and tumor growth	EGFR-TKI	157
	Cell survival, tumor growth, proliferation and metastasis	ABL2-HSF1 signaling	158
Ovarian Cancer	Proliferation	IgA anti HSF1	159, 160
	Tumor progression, cell spreading, ECM remodeling and cancer invasion	DKK3; YAP/TAZ	98, 161, 162
Endometrial Cancer	Tumor progression	ERα	163
Prostate Cancer	Tumor progression, cell survival and proliferation	Androgen receptor	164-167

**Table 4:** Current HSF1 inhibitors

Agents / compounds	Source	Effects	Cancer type	Ref.
**Natural Compounds**				
Quercetin	Plant pigment (flavonoid)	Increase apoptosis	Liver and breast cancer	178
Triptolide	*Tripterygium wilfordii*	Induce a dose-dependent increase in apoptosis	Chronic lymphocytic leukemia	179
		Downregulate proliferative pathway	Pancreatic cancer	186
		Induce cell death	Liver cancer	187
		Induce cell death	Multiple myeloma	188
2,4-Bis (4-hydroxybenzyl) phenol	*Gastrodia elata*	Induce growth arrest and apoptosis	Lung cancer	189
Cantharidin	Blister beetles (*Meloidae spp.*)	Inhibit HSP70 and BAG3 via HSF1-dependent, downregulation of antiapoptotic Bcl-2 family	Colon cancer, lung cancer, prostate cancer, breast cancer	190
**Protein Translation Inhibitors**				
Rohinitib (rocaglamide / rocaglates)	Flavaglines; found in plants of the genus Aglaia (*Meliaceae*)	Downregulates HSF1-activated genes; de-represses HSF1-repressed genes; reduce glucose uptake and lactate productions	Leukemia	191, 192
**Cyclin-Dependent Kinase Inhibitors (CDK Inhibitors)**				
SNS-032	Sulfur compounds	It is notable as the CDK inhibitors in a small-molecule screen for HSF1 inhibitors. suppressing HSP70 expression	Leukemia	193-195
4,6-disubstituted pyrimidines	Aromatic heterocyclic organic compound	Indirect HSF1 inhibitor via HSP70 and HSP90	Osteosarcoma	195
**Synthetic compounds**				
KNK437	Benzylidene lactam compound	Inhibitor of acquired thermotolerance; inhibits the AKT/HSF1, a pro-survival pathway in breast cancer; blocks HSF1-mediated transcription and induces apoptosis	Colon cancer; squamous cell carcinoma; breast cancer	196-198
NXP800 (CCT361814)	Bisamide	Direct HSF1 inhibitor led to apoptosis	Multiple myeloma; solid tumor (under clinical trial)	184, 185, 199
SISU-102(DTHIB)		Selectively stimulates degradation of nuclear HSF1	Prostate cancer; leukemia	164, 183
KRIBB11	Pyridinediamine	Induces growth arrest and apoptosis	Multiple myeloma; lung cancer	157, 172, 198, 200, 201
I_HSF1_115	Thiazole acrylamide	Pro apoptosis; inhibits the transcriptional activity	Multiple myeloma; breast cancer	198, 202
Dorsomorphin		Inhibits nuclear HSF1 accumulation	Colon cancer; prostate cancer	203
PW3405	Anthraquinone	Inhibit HSF1 phosphorylation	HeLa cancer cell	171, 198
CCT251236	Bisamide	Inhibit HSF1 transcription	Ovarian cancer	169
NZ28	Emetine	Inhibition of HSP mRNA translation; inhibit activation of HSF1 lead to inhibition of cell migration and invasion	Myeloma; prostate cancer; lung cancer; breast cancer	204, 205

## Abbreviations

ADP: Adenosine diphosphate
AML: Acute myeloid leukemia
AMPK: AMP-activated protein kinase
APC: Anaphase-promoting complex
AR: Androgen receptor
ATG7: Autophagy-related gene 7
BAG3: Bcl-2-associated athanogene-3
Bcl-2: B-cell lymphoma 2
BRCA: Breast cancer gene
CAF: Cancer-associated fibroblast
CCT: Chaperonin containing TCP-1
CDC20: Cell division cycle 20
CDK8: Cyclin-dependent kinase 8
CHIP: Carboxyl-terminus of HSP70 interacting protein
CK2: Casein kinase 2
CLL: Chronic lymphocytic leukemia
CSNK1: Casein kinase 1
DAPK: Death-associated protein kinase
DBD: DNA binding domain
DKK3: Dickkopf-related protein 3
DTHIB: Direct targeted HSF1 inhibitor
DYRK2: Dual specificity tyrosine-regulated kinase 2
ECM: Extracellular matrix
EGFR: Epidermal growth factor receptor
ER: Endoplasmic reticulum
ERα: Estrogen receptor α
ErbB2: Erythroblastic oncogene B
ERK: Extracellular signal-regulated kinase
FAM3C: Family with sequence similarity three member C
FBXW7: F-box/WD repeat-containing protein 7
FUT4: Fucosyltransferase IV
GBP: Guanylate binding protein
GPX4: Glutathione peroxidase 4
GSK3: Glycogen synthase kinase III
HCC: Hepatocellular carcinoma cell
HDAC6: Histone deacetylase 6
HER2: Human epidermal growth factor receptor 2
HIF-1α: Hypoxia-inducible factor-1α
HR: Heptad repeat
HSE: Heat shock element
HSF1: Heat shock transcription factor 1
HSP: Heat shock protein
HSPB1: Heat shock protein beta-1
HSR: Heat shock response
IFNγ: Interferon γ
IGFIIR: Insulin-like growth factor receptor II
IL-6: Interleukin 6
INHBA: Inhibin subunit beta A
JNK: c-JUN N-terminal kinase
LDHA: Lactate dehydrogenase A
LIMIT: Lnc RNA inducing MHC-1 and immunogenicity of tumor
LncRNA: Long non-coding RNA
LZ: Leucine zipper
MAPK: Mitogen-activated protein kinase
Mcl-1: Myeloid cell leukemia-1
MDR1: Multi drug resistance 1
MHC-1: Major histocompatibility complex 1
MICB: Major histocompatibility class 1 chain related-protein B
MK2: MAPK-activated protein kinase 2
mTOR: Mammalian target of rapamycin
mTORC1: mTOR complex 1
MUL1: Mitochondrial ubiquitin ligase 1
NEF: Nucleotide-exchange factor
NF-κB: Nuclear factor kappa B
p38 MAPK: p38 mitogen-activated protein kinase
PDK3: Pyruvate dehydrogenase kinase 3
PD-L1: Programmed cell death ligand protein 1
PI3K: Phosphoinositide 3-kinase
PIM2: Proviral integration site for moloney murine leukemia virus 2
PKA: Protein kinase A
PLK1: Polo-like kinase 1
PP2A: Protein phosphatase 2A
PROM2: Prominin 2
PSA: Prostate-specific antigen
p-TEFb: Positive transcription elongation factor b
PTM: Post translational modification
RD: Regulatory domain
RFA: Radiofrequency ablation
ROS: Reactive oxygen species
SDHC: Succinate dehydrogenase C
SIRT1: Sirtuin 1
SMAC: Second mitochondria-derived activator of caspase
SQSTMI: Sequestosome-1
SSBP1: Single-strand DNA-binding protein 1
SUMO: Small ubiquitin-related modifier
TAD: Transactivation domain
TBK1: TANK-binding kinase 1
TGF-β: Transforming growth factor beta
TKI: Tyrosine kinase inhibitor
TME: Tumor microenvironment
TNF-α: Tumor necrosis factor alpha
TNFR1: TNF-α receptor 1
TRiC: T-complex protein ring complex
TRIM: Tripartite motif
TRRAP: Transformation/Transcription domain associated protein
TSP2: Thrombospondin 2
TSP4: Thrombospondin 4
ULK1: Unc-51-like kinase 1
UPR: Unfolded protein response
YAP/TAZ: Yes-associated protein / Transcriptional co-activator with PDZ-binding motif
YY1: Yin Yang 1
